# Embryonal rhabdomyosarcoma of the uterine cervix in an adult female

**DOI:** 10.4322/acr.2023.419

**Published:** 2023-01-30

**Authors:** Toyaja Jadhav, Manoj Gopal Madakshira, Sushil Garud

**Affiliations:** 1 12 Airforce Hospital Department of Laboratory Sciences Gorakhpur Uttar Pradesh India 12 Airforce Hospital, Department of Laboratory Sciences, Gorakhpur, Uttar Pradesh, India; 2 Command Hospital Department of Pathology Lucknow Uttar Pradesh India Command Hospital, Department of Pathology Lucknow, Uttar Pradesh, India; 3 12 Airforce Hospital Department of Obstetrics and Gynecology Gorakhpur Uttar Pradesh India 12 Airforce Hospital, Department of Obstetrics and Gynecology, Gorakhpur, Uttar Pradesh, India

**Keywords:** Rhabdomyosarcoma, Embryonal, Uterine Cervical Diseases, Sarcoma, Cervical Cancer, Uterine Cervical Neoplasms

## Abstract

Embryonal rhabdomyosarcoma (RMS) of the female genital tract is an uncommon malignancy, presenting mainly in the pediatric and adolescent populations, primarily affecting the first two decades of life. This malignancy presentation in adulthood is rare and is seldom seen. The incidence of this tumor affecting adult females is approximately 0.4 - 1%, with the common site being the vagina. This tumor infrequently involves the cervix. RMS has a poor survival rate and once diagnosed, it requires aggressive management by radical surgery accompanied by chemoradiation. We present a case of an anaplastic variant of embryonal RMS of the uterine cervix presenting as a cervical polyp in a 36-year-old female who complained of dyspareunia and post-coital bleeding.

## INTRODUCTION

Embryonal Rhabdomyosarcoma (RMS) is a rare tumor arising from the embryonal mesenchyme and is primarily a childhood and adolescent malignancy. It is one of the most common childhood soft tissue sarcomas.^1^ It accounts for 4 to 6% of all malignancies in this age group, while in adults, it comprises less than 1% of all malignancies.^1^^,^^2^ RMS commonly affects the head and neck region, while the female genital tract remains the second most common presentation site. Other affected sites include the extremities, the trunk, and the retroperitoneum, apart from other uncommon sites reported in the literature, such as the thorax, gastrointestinal tract (GIT), anus, and perianal regions.^3^ In the female genital tract, the vagina is the most commonly affected site, and embryonal RMS commonly presents as botryoid RMS in the vagina. The uterine cervix is an uncommon site for the development of embryonal RMS, and this tumor comprises 0.2% of the malignant tumors of the uterus affecting adult females. The ratio of incidence of cervical primary embryonal RMS compared to its vaginal counterpart is 1:5, while the survival rates are known to be 60% and 90%, respectively.^3^^,^^4^ The diagnosis of this variant rests primarily upon clinical suspicion clubbed with appropriate histopathological and immunohistochemical evaluation. Since these tumors are highly infrequent among adults, they can be easily missed or misdiagnosed in the absence of a green eye.

The classification of rhabdomyosarcoma subtypes is critical in terms of prognosis and therapeutic definition. Generally, alveolar and pleomorphic subtypes have a poor prognosis, but the embryonal subtype has a relatively better prognosis. Morphological and molecular characteristics will be discussed to support risk stratification and therapeutic choice. We present a case of an anaplastic variant of RMS presenting as a cervical polyp in a 36-year- old female

## CASE REPORT

A 36-year-old female presented to the Gynecology Out Patient Department (OPD) with chief complaints of dyspareunia and post-coital bleeding. The gynecological evaluation revealed the presence of a large cervical polyp arising from the endocervix, which bled on touch. She had no other significant co-morbidity and was worked up for the polyp’s surgical excision. Intraoperatively, disproportional bleeding followed the polyp’s removal.

**Figure 1:**
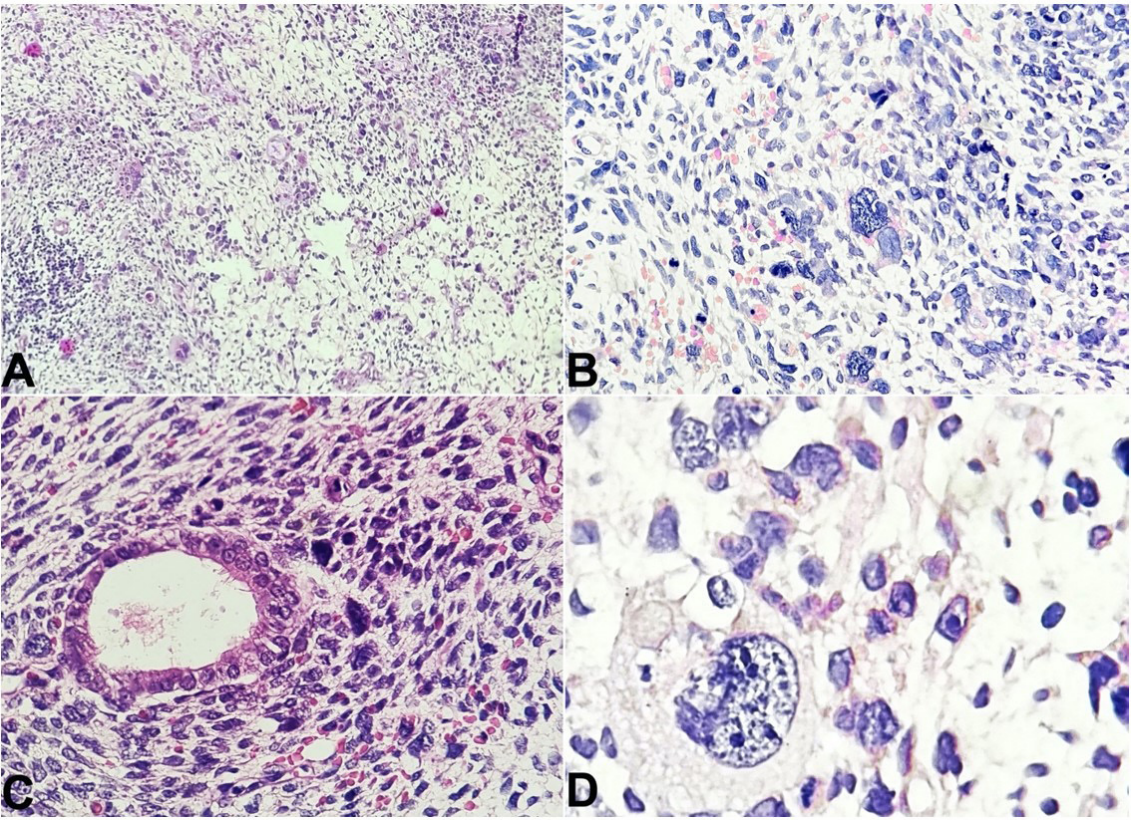
Photomicrographs of the cervical polyp. A - Scanner view shows a stromal tumor composed of marked cytological atypia in the form of nuclear pleomorphism, high N:C ratio, and markedly pleomorphic cells with scant cytoplasm and bizarre nuclei, with the presence of binucleation and fetoid cells with conspicuous nucleoli (H&E; 40x); B - shows the presence of nuclear pleomorphism and atypia with the presence of fetoid/embryonal cells and atypical mitotic figures (H&E; 100x); C - shows stromal tumor cells surrounding an endometrial gland (H&E; 100x); D - shows a fetoid tumor cell with nuclear irregularity, high N:C ratio and presence of multiple conspicuous nucleoli present among surrounding tumor cells (H&E; 400x).

The polyp measured 5x3cm and presented a blood-stained external surface. It was fully processed. The histological examination revealed a stromal tumor (Figure 1) composed of marked cytological atypia with nuclear pleomorphism, high N:C ratio, and markedly pleomorphic cells with scant cytoplasm and bizarre nuclei, with the presence of binucleation and undifferentiated atypical blastemal cells with conspicuous nucleoli.

Brisk mitosis was noted with 1-2 atypical mitotic figures per high-power field (1-2/HPF). A few strap cells were also present scattered within the tumor cells. The tumor cells encroached upon the blood vessels, but no frank vascular invasion was noted. Areas of hemorrhage and necrosis were also present. Atrophic endometrial glands were also found within the tumor.

The overall microscopy of the polyp connoted a diagnosis of a high-grade genitourinary stromal neoplasm with the differentials being (i) pleomorphic undifferentiated uterine sarcoma, (ii) high-grade endometrial stromal sarcoma, (iii) dedifferentiated cervical leiomyosarcoma, and (iv) rhabdomyosarcoma.

**Figure 2:**
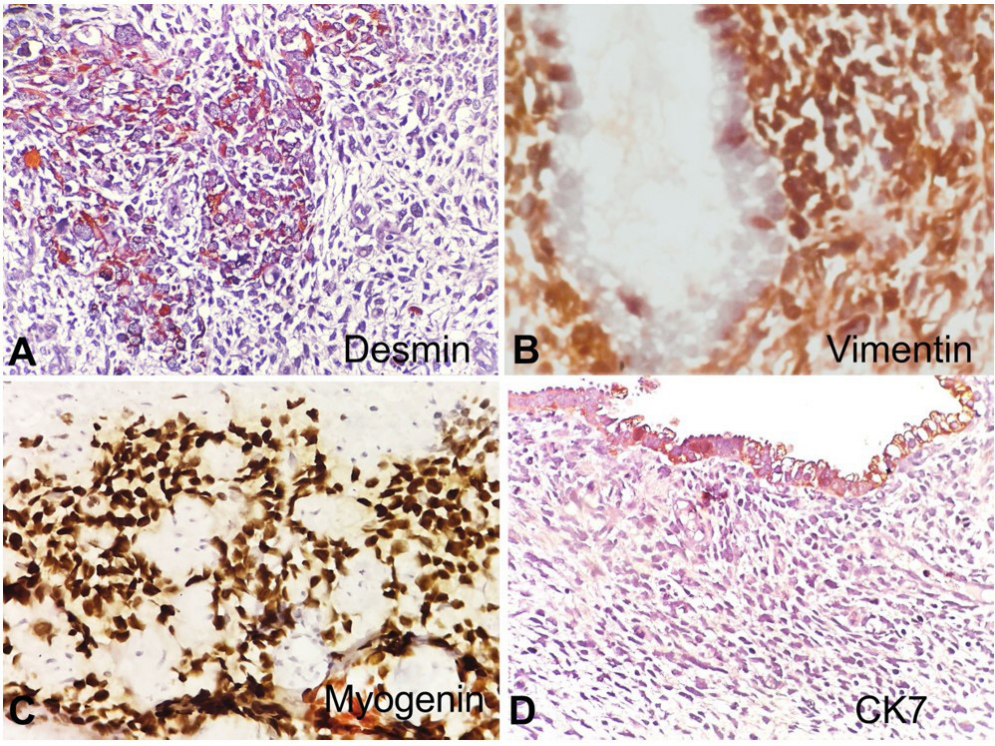
Photomicrographs of the cervical polyp. A - shows immunoreactivity to Desmin (100x); B - Vimentin (100x), and C - Myogenin (100x); D - negative reaction to CK7 (100x).

Immunohistochemical (IHC) evaluation revealed tumor cell reactivity to Vimentin, Desmin, Myo D1, and Myogenin but was non-reactive for SMA and CK7 (Figure 2). The Ki 67 proliferative index was 50-60% in the most proliferative focus.

The final microscopy with IHC studies established a diagnosis of Embryonal RMS of the uterine cervix, an anaplastic variant.

**Figure 3:**
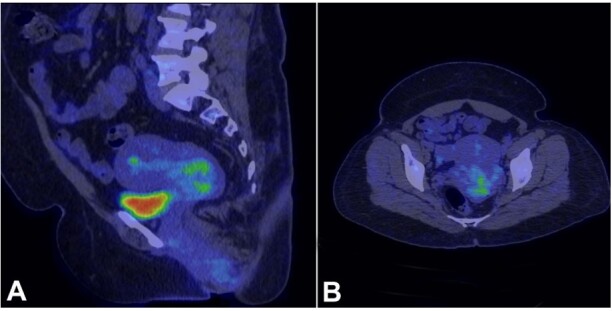
PET CT of the patient shows in A (sagittal) and B (axial) - a well-defined, slightly lobulated non-FDG avid lesion within the endocervical canal involving the posterior lip of the cervix and extending into the posterior cervical myometrium measuring 3.9x4x3.3cm, without invading into the uterus, urinary bladder, and the surrounding lymph nodes.

The patient was referred to a higher oncology center for further evaluation and management. Her PET CT revealed a 3.9x4x3.3cm, non-FDG avid lesion involving the posterior lip and the body of the cervix (Figure 3).

Similarly, the contrast-enhanced MRI (CE-MRI) scan concurred with the above findings by confirming the presence of an endocervical canal lesion having a lobulated appearance, extending into the posterior fornix and penetrating the posterior cervical myometrium. This lesion measured 28x59x47mm. However, no invasion into the uterus or the urinary bladder was noted.

Based on the above radiological features, the patient underwent total abdominal hysterectomy with bilateral salpingo-oophorectomy with bilateral pelvic lymph node dissection (TAH+BSO+B/L PLND), and the excised specimens were evaluated histopathologically.

**Figure 4:**
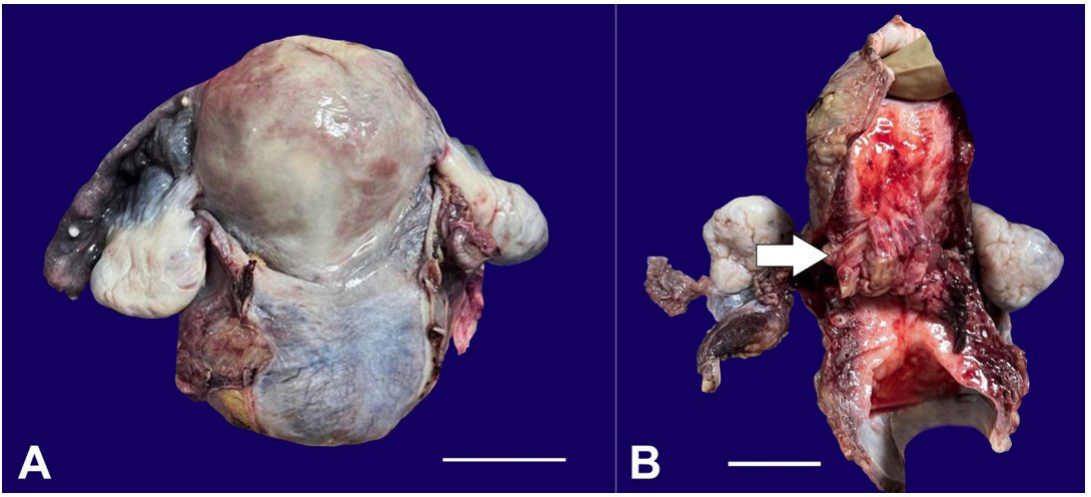
Gross image of the resected uterus after TAH+BSO with the external surface of the uterus and cervix appearing smooth (A) (scale bar= 10 cm); and the cut surface showing a cervical tumor (arrow), extending into the lower uterine segment (B) (scale bar= 10cm).

Grossly, the uterus presented with an exophytic polypoidal growth measuring approximately 6x4x2.5cm, arising from the endocervical canal with unremarkable involvement of the remaining endometrial cavity (Figure 4A and 4B). The endometrial thickness was 1.6 cm. The microscopy of the polypoidal mass concorded with the initial biopsy findings and the presence of a cambium layer, with tumor cells showing focal rhabdomyoblastic differentiation and brisk mitosis. Although IHC features were the same as on the initial biopsy, the Ki67 proliferation index of this tumor was slightly higher at 60-70% in most proliferative areas.

The final histopathological diagnosis of the TAH+BSO+B/L PLND specimen concluded to be cervical embryonal rhabdomyosarcoma, an anaplastic variant with no lymphovascular invasion (LVI) or perineural invasion (PNI) or extension into the myometrium or the adjacent Fallopian tubes or ovaries with the dissected lymph nodes being free of tumor.

The patient is currently undergoing chemoradiotherapy and is on regular follow-up.

## DISCUSSION

RMS is a highly malignant skeletal muscle neoplasm. Horn and Enterline^5^ initially classified it into alveolar, embryonal, the botryoid variant of embryonal and pleomorphic RMS; however, in 2013, the WHO classification^6^ of skeletal muscle tumors modified the RMS histologic classification, describing sclerosing RMS as a separate entity, and not a subtype of embryonal RMS. The current WHO classification subclassifies RMS into embryonal, alveolar, spindle cell/sclerosing, and pleomorphic variants and does not separate the botryoid subtype.^2^^,^^6^^,^^7^ In recent years, anaplastic or pleomorphic and embryonal RMS have emerged as the two main subtypes of RMS based on the microscopic features of the cells distributed around a central area or cells resembling immature skeletal myoblasts. This distinction is supported by the recognition that the anaplastic variant is often associated with balanced chromosomal translocations involving chromosomes 2 or 1 and 13 (referred to here as t(2;13) and t(1;13)), originally detected by cytogenetics. However, a small but substantial fraction of patients with this variant do not harbor one of these translocations, and these tumors are biologically and clinically similar to embryonal RMS.^8^ Additionally, pleomorphic RMS is a morphologically uncommon variant of RMS that typically occurs in adults. Like the embryonal variant, unifying molecular genetic aberrations in pleomorphic RMS are unclear. The spindle cell/sclerosing RMS variant is more common in children. The tumors in the head/neck region seem more likely to carry specific somatic mutations and have a poorer prognosis.^9^

Embryonal RMS is the most common type and accounts for 60% of all rhabdomyosarcomas.^7^ It is a malicious variant of RMS and arises from the embryonal mesenchyme. It accounts for 4-6% of all childhood malignancies.^7^ It is further categorized into sarcoma botryoides and anaplastic variants. The anaplastic variant is an uncommon variant of embryonal RMS and comprises roughly 8-27% of all embryonal RMS.^10^^,^^11^ It is primarily a childhood malignancy and is almost always associated with TP53 mutations and a poor prognostic outcome.^12^ The 2008 and 2021 reports from Soft Tissue Sarcoma Committee for the Children’s Oncology Group mentioned that embryonal RMS most commonly presented in the 1^st^ decade of life, with approximately 82% of these cases having *TP53* mutations.^10^^,^^11^

Although rhabdomyosarcoma is considered relatively common among children with vaginal sarcoma, embryonal cervical rhabdomyosarcoma is extremely rare and usually occurs in women in the second and third decades of life.^13^ Approximately 1% of all adult malignancies are sarcomas, and rhabdomyosarcomas account for less than 5% of all adult sarcomas.^14^ As per the literature review of this malignancy given by Connor and Disilvestro^12^ in 2015, there have been less than 40 reported cases of cervical embryonal rhabdomyosarcoma in adult women in the last 50 years. Additionally, of all the malignancies affecting the uterine cervix, the incidence of RMS is reported to be about 0.4 to 1%.^15^

Most cervical RMS cases are known to occur sporadically, with no recognized predisposing factors; however, a small proportion of cases are also associated with genetic conditions. Li-Fraumeni cancer susceptibility syndrome, evident by a clustering of soft tissue malignancies (including sarcomas), has been discovered in a family to be caused by a heterozygous germline p53 mutation.^16^ It has also recently been associated with constitutional mismatch repair-deficiency syndrome and dysplasia syndrome. Dehner et al.^14^ in their study of 14 cases of cervical embryonal RMS, also found a link of embryonal RMS to the pleuropulmonary blastoma family of tumors with confirmed *DICER1* mutations.

The pathogenesis of embryonal RMS of the cervix remains unclear; however, several reports implicate germline mutations involving the *DICER1* gene. The *DICER1* gene codes for endoribonuclease, which has an important role in the biogenesis of microRNAs and the control of protein translation.^17^ Apellaniz-Ruiz et al.^16^ in their study of embryonal RMS and adenosarcomas of the gynecological tract, have observed that almost all cases of gynecologic embryonal RMS may harbor *DICER1* alterations. This pathological germline variation in *DICER1* may create a predisposition to hereditary cancer syndrome - DICER1 syndrome, characterized by developing multiple benign and malignant tumors. This presents a similarity with the study of Dehner et al.^14^ where they linked the development of cervical embryonal RMS to the blastoma family of tumors with *DICER1* mutation. Additionally, inactivating mutations of the p53 tumor suppressor gene located on chromosome 17 have also been described.^17^^,^^18^

In the cervix, embryonal RMS is known to predominantly present as a cervical polyp, commonly associated with bleeding, as was our patient’s presentation. The initial clue for diagnosing our case on gross evaluation was the size of the polyp and its gross appearance. Cervical RMS often presents as a large polyp with many myxoid areas. It may also present as multiple polyps.^2^ Our case did present with a large polyp; however, its surface was blood-stained, so the presence of myxoid areas could not be ascertained grossly.

The clinical manifestations of cervical RMS include serosanguinous vaginal discharge and a polyp-like mass protruding through the vagina.^2^ Our patient, similarly, presented with postcoital bleeding. Other clinical symptoms include amenorrhea or menorrhagia.^19^ Advanced cases may present with cervical pain radiating to the back, bladder irritation, and tenesmus. Terminal stages of the malignancy give rise to anemia and cachexia and may even develop hydronephrosis.^2^

Microscopically, it is characterized by hypocellular and hypercellular areas with a loose, myxoid stroma. Perivascular condensations of tumor cells in the less cellular regions are common. Marked pleomorphism is noted, along with abundant atypical mitosis and atypical rhabdomyoblasts. Immunohistochemistry reveals tumor cells’ reactivity to vimentin, desmin, actin, myoglobin, MyoD1, and myogenin. IHC is essential for a confirmatory diagnosis.^19^^,^^20^

Histopathologically, this tumor needs to be differentiated from other benign and malignant mimics such as cervical mesodermal polyp (pseudosarcoma botryoides), rhabdomyoma, cervical adenosarcoma, pleomorphic undifferentiated uterine sarcoma, high-grade endometrial stromal sarcoma, and dedifferentiated cervical leiomyosarcoma.

Tables 1 and 2 differentiate embryonal RMS from its benign and malignant mimics, respectively.

**Table 1:** Comparison of Embryonal RMS with its benign mimics

**Feature**	**Rhabdomyoma (RM) Adult and Genital types**	**Pseudosarcoma botryoides**	**Embryonal RMS**
Behavior^21^	Benign	Benign	Malignant
Differentiation^20^^,^^22^^-^^24^	Skeletal muscle	Skeletal muscle	Skeletal muscle
Age group commonly affected^22^^-^^26^	Adult type: Median age 60 y Genital type: Median age 42 y	Median age 35 y	1^st^ decade of life
M:F ratio^20^^,^^22^^-^^25^^,^^27^	Adult type = 3:1 Genital type = Female predominance	Female predominant.	Conventional embryonal RMS is more common among males. Cervical RMS is female predominant
Common site of presentation^22^^-^^25^^,^^27^^,^^28^	Adult type = Oropharynx, larynx and muscles of the neck. Genital type = Vulva and vagina	Vagina. May also occur in the vulva and cervix	Head, neck and genitourinary system
Etiopathogenesis^29^^-^^31^	Adult RM is of clonal origin. Genital RM: Hedgehog signaling (SHH pathway activation) and association with Gorlin syndrome	02 hypothesis indicated: Reactive hyperplastic process of subepithelial stroma. Likely hormonal role since stromal cells of FSPs express ER and the PR.	Overactive IGF2 gene on chromosome 11, promoting tumorigenesis
Gross^20^^,^^22^^,^^23^^,^^25^	Adult RM- round or polypoid mass in the neck region. Genital type presents as a genital polyp covered by smooth mucosa.	Pedunculated or not polypoid masses	Poorly circumscribed mass, white, soft or firm, infiltrative
Microscopy^20^^,^^22^^-^^25^	Adult RM - well circumscribed, unencapsulated sheets of large well-differentiated round to polygonal skeletal muscle cells with abundant eosinophilic granular cytoplasm with frequent loss of striations and presence of cytoplasmic rod like inclusions and small, round and vesicular nuclei with or without prominent nucleoli. Genital RM - haphazard strap like muscle fiber cells with a central vesicular uniform nucleus and abundant eosinophilic cytoplasm in a background of fibrous stroma and dilated blood vessels. Rare mitosis seen.	Variable stromal cellularity with cytologic atypia composed of stellate cells, and multinuclear cells	Composed of primitive mesenchymal cells that show variable degrees of skeletal muscle differentiation with marked cytological atypia and presence of undifferentiated or fetoid cells in a background of hypocellular and hypercellular, often myxoid stroma.
Immunohistochemistry^20^^,^^22^^-^^25^	Adult and genital RM: SMA +ve; Desmin +ve; Myoglobin +v”; EMA -ve, CD68 -ve and CK -ve Adult RM are also PAS diastase positive and may show a weak expression of S100.	ER +ve PR +ve Vimentin +ve Desmin +ve CD10 +ve SMA +ve	Desmin +ve Vimentin +ve Myo D1 +ve Myogenin +ve
Management^21^^,^^25^^,^^32^	Surgical resection	Simple excision	Depends on the location; mainstay remains surgical tumor excision followed by chemotherapy and/or radiotherapy
Prognosis^21^^,^^25^^,^^30^^,^^33^	Good	Good	Age group of 5-9 years have the best prognosis, those <1 year and >10 years have a poor survival outcome.

**Table 2:** Comparison of Embryonal RMS with its malignant mimics

**Features**	**Embryonal RMS**	**Undifferentiated Uterine sarcoma**	**High-grade endometrial stromal sarcoma**	**Dedifferentiated Leiomyosarcoma**	**Adenosarcoma**
Behavior	Malignant	Malignant	Malignant	Malignant	Mixed
Differentiation^34^^-^^38^	Skeletal muscle	Mesenchymal tumor with no specific lineage	Endometrial stroma	Uterine myometrium	Benign epithelial component with malignant stromal component
Age group most commonly affected^33^^,^^35^^-^^39^	1^st^ decade of life and adolescence	Post - menopausal; median age 7^th^ decade	Mostly 4^th^ -5^th^ decade of life	Post-menopausal	Peri and post-menopausal. Mean age is 50 years
M:F ratio^27^^,^^35^^,^^36^^,^^38^^,^^39^	Conventional embryonal RMS predominates males, genital RMS - females	Female predominant	Female predominant	Female predominant	Female predominant
Common site of presentation^20^^,^^35^^,^^36^^,^^38^^,^^40^	Head and neck region	Uterus	Uterine corpus	Retroperitoneum	Commonly in Uterine corpus Sites of previous endometriosis: Cervix, ovary, fallopian tube, vagina, peritoneum
Etio -pathogenesis^20^^,^^35^^,^^36^^,^^38^^,^^41^	Cervical Embryonal RMS: Germline mutations involving the DICER1 gene Conventional embryonal RMS: Overactive IGF2 gene on chromosome 11, promoting tumorigenesis. Also associated with defects in RAS or Hedgehog pathways	Overexpression of specific genes	Genetic alterations such as YWHAE-NUTM2A/B fusions, BCOR fusions, BCOR ITD and high-grade transformation of low grade endometrial stromal sarcoma	Primary etiology is unknown. It is mostly associated with EBV in HIV positive patients.	Endometriosis, previous pelvic radiotherapy, long term unopposed estrogen therapy and tamoxifen use may be risk factors. Pathogenesis remains largely unknown.
Gross^20^^,^^35^^,^^36^^,^^38^^,^^41^	Poorly circumscribed mass, white, soft or firm, infiltrative	Generally, a large tumor with fleshy, pink-tan cut surface with surface hemorrhage and necrosis.	Soft, fleshy yellow or tan pink polypoid mass, rubbery in consistency.	Large, soft masses, often with necrosis, hemorrhage and cystic degeneration. Osseous differentiation may present with bony foci.	Usually forms polypoid masses ranging from 1 - 20 cm. Typically fills the endometrial cavity and may project into the endocervical canal. Cut surface is solid, white to tan in color with multiple small soft and fleshy cysts containing watery or mucoid fluid.
Microscopy^20^^,^^35^^-^^38^	Composed of primitive mesenchymal cells that show variable degrees of skeletal muscle differentiation with marked cytological atypia and presence of undifferentiated or fetoid cells in a background of hypocellular and hypercellular, often myxoid stroma.	Spindle, epithelioid or polygonal cells with marked nuclear atypia and pleomorphism, frequent multinucleation and macronucleoli.	Permeative tumors with vaguely nested growth of round cells with scant to moderate eosinophilic cytoplasm and uniform nuclear atypia with presence of brisk mitosis associated with LVI and tumour necrosis.	Low-grade to high grade leiomyosarcoma associated with a discrete undifferentiated component lacking morphological or immunophenotypic features of myogenic differentiation	Biphasic tumor with admixed glands and prominent stroma showing marked atypia.
IHC features^17^^,^^34^^-^^36^^,^^38^^,^^39^^,^^41^^-^^43^^,^^41^^,^^44^	Desmin +ve Vimentin +ve Myo D1 +ve Myogenin +ve	Nonspecific CD10+ve p53 +ve in 50% cases Focal SMA +ve Strong p16 may be seen ER and PR -ve	CD10+ve Cyclin D1 +ve BCOR +ve ER/PR +ve Desmin -ve SMA -ve	HHF 35, αSMA, Vimentin, Desmin, H-Caldesmon positivity in the low to high grade Leiomyosarcoma component, while the dedifferentiated component may not show any of the myogenic marker reactivity.	Glandular component shows AE1 / AE3, ER and PR; variable CD10 reactivity; while the mesenchymal component shows vimentin, CD10, WT1, ER and PR reactivity with variable reactivity to SMA. Desmin, CD34 and Cytokeratin
Management.^32^^,^^42^^-^^45^	Chemotherapy, radiation therapy and surgery	Hysterectomy with bilateral salpingo- oophorectomy with adjuvant radio and/or chemotherapy	Chemotherapy after surgery	Surgery aiming at complete resection.	Surgery is the primary treatment; chemotherapy can be somewhat beneficial
5-year survival^46^^,^^47^	27% (in adults) 61% (children)	43%	Stage I -90% Stage III - IV -50%	20-30%	79% for early stage 48% for stage III cases

Due to uncommon occurrences of these tumors, there is limited literature evaluating optimal therapy. Hence, there is no uniform consensus on the management approach to these tumors. However, over the decades, there has been a paradigm shift in management strategies for cervical embryonal RMS. Although ultra-radical surgeries like pelvic exenteration were considered the treatment of choice in the late 1960s, outcomes were often unsatisfactory. In the 1970s, limited surgery with adjuvant chemotherapy and/or irradiation showed improved survival. Surgical aggressiveness has gradually reduced from mutilating exenterative procedures to simple local excisions.^48^

The current management of cervical embryonal RMS depends primarily on the patient’s age. Since it is more common among younger females, conservative management to retain the genitourinary organs is the preferred modality. Other factors taken into account are the histologic subtype, size, site of origin, disease extent at presentation, and residual disease after treatment. Since cervical RMS is less aggressive than its vaginal counterpart, fertility-sparing options can be considered for managing the cervix’s early stage of embryonal RMS. The Intergroup Rhabdomyosarcoma Study Group (IRSG) states fertility-sparing surgery and chemotherapy as an appropriate treatment for patients with localized disease but not applicable for advanced-stage and metastatic disease. The primary principle for surgical management of cervical embryonal RMS is complete resection of the primary tumor with the surrounding margin of normal tissue.^7^

The spectrum of surgical therapy now includes radical hysterectomy with or without lymphadenectomy, vaginectomy, cervicectomy, polypectomy, local excisions, and diathermy loop excisions. Embryonal RMS of the cervix are mostly either treated with surgery alone or with adjuvant chemotherapy and/or radiotherapy.^48^

The literature indicates regional lymph node removal as an option in patients presenting with low-risk disease manifestations but a recommended compulsion in patients with high-risk disease.^7^

Additionally, RMS is also known to be a chemosensitive tumor. Adjuvant chemotherapy targets micro lymphatic metastases if any. Even with adjuvant chemotherapy regimens, there is no uniform agreement in managing these tumors. Most investigators have used a combination of two or three chemotherapeutic agents. The most widely used chemotherapy regimen is VAC (Vincristine -Actinomycin- Cyclophosphamide), which is the current gold standard chemotherapy regimen utilized for the management of cervical embryonal RMS, including botryoid RMS.^48^

Trials with Ifosfamide also proved successful as a chemotherapeutic agent in the management of cervical RMS, in combination with vincristine and actinomycin (VIA regimen). However, in Intergroup Rhabdomyosarcoma Study - IV (IRS IV), patients were randomized to receive chemotherapy with VAC or VAI. No significant difference in outcome was noted, and the American investigators elected the VAC as the gold-standard due to the lower cost and nephrotoxicity of cyclophosphamide.^7^

The survival rate in patients with cervical embryonal RMS depends upon a number of factors, where (i) the tumor stage and (ii) the modality of management are important determining factors. Most patients can survive disease-free for more than 12 months for early-stage malignancy through surgery, radiotherapy, and chemotherapy. Additionally, research has shown that for adult rhabdomyosarcoma, surgery combined with VAC chemotherapy can have an overall survival of 55% and a disease-free survival rate of 64% at 2 years.^14^^,^^49^

## CONCLUSION

Embryonal RMS of the uterine cervix is a rare malignancy in adults with a relatively poor prognostic outcome. Although rare, it should be suspected in a patient with vaginal bleeding and a large cervical polyp. Histopathology combined with appropriate immunohistochemistry plays a major role in the pathological detection of the tumor and, thereby, planning apposite management. Timely detection of this rare adult tumor can go a long way in improving the patient's survival.

## References

[1] 1 Jayi S, Bouguern H, Fdili FZ, et al. Embryonal rhabdomyosarcoma of the cervix presenting as a cervical polyp in a 16-year-old adolescent: A case report. J Med Case Rep. 2014;8(1):241. 10.1186/1752-1947-8-241. PMid:24986146. PMC4092352 24986146

[2] 2 Shim AR, Lee M, Paek JH, Kim MJ, Kim SW. A case of embryonal rhabdomyosarcoma of the uterine cervix in a middle-aged woman. Korean Journal of Obstetrics & Gynecology. 2011;54(11):707. 10.5468/KJOG.2011.54.11.707.

[3] 3 Gomes AR, Leite PB, Rocha JS, et al. Embryonal rhabdomyosarcoma of the uterine cervix: a rare case report. Int J Reprod Contracept Obstet Gynecol. 2016;6(1):309. 10.18203/2320-1770.ijrcog20164683.

[4] 4 Mousavi A, Akhavan S. Sarcoma botryoides (embryonal rhabdomyosarcoma) of the uterine cervix in sisters. J Gynecol Oncol. 2010;21(4):273-5. 10.3802/jgo.2010.21.4.273. PMid:21278891. PMC3026308 21278891

[5] 5 Horn RC Jr, Enterline HT. Rhabdomyosarcoma: a clinicopathological study and classification of 39 cases. Cancer. 1958;11(1):181-99. 10.1002/1097-0142(195801/02)11:1<181::AID-CNCR2820110130>3.0.CO;2-I. PMid:13500314. 13500314

[6] 6 Rudzinski ER, Anderson JR, Hawkins DS, Skapek SX, Parham DM, Teot LA. The world health organization classification of skeletal muscle tumors in pediatric rhabdomyosarcoma a report from the children’s oncology group. Arch Pathol Lab Med. 2015;139(10):1281-7. 10.5858/arpa.2014-0475-OA. PMid:25989287. PMC4651658 25989287

[7] 7 Buruiana FE, Gupta B, Singh K. Rhabdomyosarcoma of the cervix in teenagers - Is fertility preservation a feasible option? Gynecol Oncol Rep. 2020;34:100677. 10.1016/j.gore.2020.100677. PMid:33304979. PMC7708689 33304979

[8] 8 Qualman S, Lynch J, Bridge J, et al. Prevalence and clinical impact of anaplasia in childhood rhabdomyosarcoma: a report from the Soft Tissue Sarcoma Committee of the Children’s Oncology Group. Cancer. 2008;113(11):3242-7. 10.1002/cncr.23929. PMid:18985676. PMC2727712 18985676

[9] 9 Shenoy A, Alvarez E, Chi YY, et al. The prognostic significance of anaplasia in childhood rhabdomyosarcoma: a report from the Children’s Oncology Group. Eur J Cancer. 2021;143:127-33. 10.1016/j.ejca.2020.10.018. PMid:33302115. PMC8842073 33302115

[10] 10 Cramer SL, Miller AL, Pressey JG, et al. Pediatric anaplastic embryonal rhabdomyosarcoma: targeted therapy guided by genetic analysis and a patient-derived xenograft study. Front Oncol. 2018;7(JAN):327. 10.3389/fonc.2017.00327. PMid:29376028. PMC5768639 29376028

[11] 11 Kaseb H, Kuhn J, Babiker HM. StatPearls. Treasure Island (FL): StatPearls Publishing; 2022.

[12] 12 Connor EV, Disilvestro PA. Obstetrics and gynaecology cases-reviews diagnosis and management of embryonal rhabdomyosarcoma in a woman with prolapsing cervical mass. Obstet Gynecol Cases Rev. 2015;2(4):4. 10.23937/2377-9004/1410050.

[13] 13 Khosla D, Gupta R, Srinivasan R, Patel FD, Rajwanshi A. Sarcomas of uterine cervix: clinicopathological features, treatment, and outcome. Int J Gynecol Cancer. 2012;22(6):1026-30. 10.1097/IGC.0b013e31825a97f6. PMid:22740005. 22740005

[14] 14 Dehner LP, Jarzembowski JA, Hill DA. Embryonal rhabdomyosarcoma of the uterine cervix: a report of 14 cases and a discussion of its unusual clinicopathological associations. Mod Pathol. 2012;25(4):602-14. 10.1038/modpathol.2011.185. PMid:22157934. PMC5031244 22157934

[15] 15 Bahall V, de Barry L, Sankar S. A rare case of embryonal rhabdomyosarcoma of the uterine cervix. Case Rep Pathol. 2022;2022:8459566. 10.1155/2022/8459566. PMid:35464884. PMC9020987 35464884

[16] 16 Apellaniz-Ruiz M, McCluggage WG, Foulkes WD. DICER1‐associated embryonal rhabdomyosarcoma and adenosarcoma of the gynecologic tract: pathology, molecular genetics, and indications for molecular testing. Genes Chromosomes Cancer. 2021;60(3):217-33. 10.1002/gcc.22913. PMid:33135284. 33135284

[17] 17 Clay MR. Soft tissue; Skeletal muscle Rhabdomyosarcoma; Embryonal rhabdomyosarcoma. [place unknown]; 2022 [cited 2022 Oct 22]. Available from: https://www.pathologyoutlines.com/topic/softtissueembryonalrhabdo.html

[18] 18 Ibrahim U, Saqib A, Mohammad F, et al. Embryonal rhabdomyosarcoma of the cervix: a rare disease at an uncommon age. Cureus. 2017;9(11):e1864. 10.7759/cureus.1864. PMid:29375950. PMC5773277 29375950

[19] 19 Meng L, Zhang Q, Han Q, Sun X, Liu Y, Huang X. Embryonic cervical rhabdomyosarcoma complicated with uterine inversion with cerebral venous sinus thrombosis as the first symptom: a case report and literature review. J Int Med Res. 2021;49(8):3000605211031776. 10.1177/03000605211031776. PMid:34369193. PMC8358511 34369193

[20] 20 Gibson MC, Nasiri N. Rhabdomyoma. Vienna: WikiDoc; 2019 [cited 2023 Jan 4]. Available from: https://www.wikidoc.org/index.php/Rhabdomyoma

[21] 21 Elliott G, Reynolds H, Fidler H. Pseudo-sarcoma botryoides of cervix and vagina in pregnancy*. J Obstet Gynaecol Br Commonw. 1967;74(5):728-33. 10.1111/j.1471-0528.1967.tb03787.x. PMid:6058536. 6058536

[22] 22 Heller A, Ukazu A, Wang Q. Pseudosarcomatous vaginal polyp: a benign mimic of malignancy. Int J Surg Pathol. 2016;1(2):1-2. 10.1177/1066896916666676 27571791

[23] 23 Lindberg M, Lucas D, Gardner JM, Cassarino D, Stallings-Archer K. Diagnostic pathology: soft tissue tumours. 2nd ed. Local: Elsevier; 2016. p. 372-7: Embryonal Rhabdomyosarcoma.

[24] 24 Narayana Kurup JK, Kamble VC, Acharya AM, Bhat AK. Massive embryonal rhabdomyosarcoma of the hand in an infant with metastasis at birth: management dilemma. Hand (N Y). 2017;12(5):NP109-12. 10.1177/1558944716685827. PMid:28718313. PMC5684939 28718313

[25] 25 Norris HJ, Taylor HB. Polyps of the vagina. A benign lesion resembling sarcoma botryoides. Cancer. 1966;19(2):227-32. 10.1002/1097-0142(196602)19:2<227::AID-CNCR2820190214>3.0.CO;2-W. PMid:5905466. 5905466

[26] 26 Health Jade. Rhabdomyoma [place unknown]; c2019 [cited 2023 Jan 4]. Available from: https://healthjade.net/rhabdomyoma/

[27] 27 Song JS, Song DE, Kim KR, Ro JY. Cellular pseudosarcomatous fibroepithelial stromal polyp of the vagina during pregnancy: a lesion that is overdiagnosed as a malignant tumor. Korean J Pathol. 2012;46(5):494-8. 10.4132/KoreanJPathol.2012.46.5.494. PMid:23136578. PMC3490121 23136578

[28] 28 Wyant T, Alteri R, Kalidas M, et al. Rhabdomyosarcoma causes, risk factors, and prevention[place unknown]; 2018 [cited 2022 Oct 22]. Available from: https://www.cancer.org/content/dam/CRC/PDF/Public/8804.00.pdf

[29] 29 National Cancer Institute. Childhood Rhabdomyosarcoma Treatment (PDQ®)-Patient Version. [place unknown]; 2022 [cited 2022 Oct 22]. Available from: https://www.cancer.gov/types/soft-tissue-sarcoma/hp/adult-soft-tissue-treatment-pdq

[30] 30 Wang X, Feng J, Li Z, Zhang X, Chen J, Feng G. Characteristics and prognosis of embryonal rhabdomyosarcoma in children and adolescents: an analysis of 464 cases from the SEER database. Pediatr Investig. 2020;4(4):242-9. 10.1002/ped4.12220. PMid:33376951. PMC7768301 33376951

[31] 31 Caruso RA, Napoli P, Villari D, Starrantino M. Anaplastic (pleomorphic) subtype embryonal rhabdomyosarcoma of the cervix. Arch Gynecol Obstet. 2004;270(4):278-80. 10.1007/s00404-003-0504-y. PMid:12942264. 12942264

[32] 32 Chapell DB. Uterus: other mesenchymal tumors - Undifferentiated uterine sarcoma. [place unknown]; 2021 [cited 2022 Oct 22]. Available from: https://www.pathologyoutlines.com/topic/uterussarcoma.html

[33] 33 Han L, Benett J. Uterus Stromal tumors: high grade endometrial stromal sarcoma. [place unknown]; 2021 [cited 2022 Oct 22]. Available from: https://www.pathologyoutlines.com/topic/uterusESShighgrade.html

[34] 34 Rawish KR, Fadare O. Dedifferentiated leiomyosarcoma of the uterus with heterologous elements: a potential diagnostic pitfall. Case Rep Obstet Gynecol. 2012;2012:534634. 10.1155/2012/534634. PMid:23119198. PMC3483660 23119198

[35] 35 Özen ÖI, Ayhan A. Uterus: mixed epithelial and mesenchymal tumors - Müllerian adenosarcoma. [place unknown]; 2021 [cited 2022 Oct 22]. Available from: https://www.pathologyoutlines.com/topic/cervixadenosarcoma.html

[36] 36 Iihara K, Hirano K, Fujioka Y, Sakamoto A. Leiomyosarcoma with dedifferentiation in a premenopausal patient discovered after uterine artery embolization. Pathol Int. 2007;57(10):681-7. 10.1111/j.1440-1827.2007.02157.x. PMid:17803657. 17803657

[37] 37 Nicolas MM, Tamboli P, Gomez JA, Czerniak BA. Pleomorphic and dedifferentiated leiomyosarcoma: clinicopathologic and immunohistochemical study of 41 cases. Hum Pathol. 2010;41(5):663-71. 10.1016/j.humpath.2009.10.005. PMid:20004935. 20004935

[38] 38 Shankar V. Soft tissue: smooth muscle - Leiomyosarcoma; Leiomyosarcoma-general[place unknown]; 2022 [cited 2022 Oct 22]. Available from: https://www.pathologyoutlines.com/topic/softtissueleiomyosarcoma.html

[39] 39 Allanson ER, Cummings M, Kuchel A, Wastney T, Nicklin J. Undifferentiated uterine sarcoma: a multidisciplinary challenge. Int J Gynecol Cancer. 2022;32(2):198-202. 10.1136/ijgc-2021-003215. PMid:35131912. 35131912

[40] 40 Zhang Y, Chen C, Ren M, Cong X, Li Z, Yang L. Treatment of uterine high-grade endometrial stromal sarcoma with apatinib combined with chemotherapy: a case report. Medicine (Baltimore). 2019;98(13):e15050. 10.1097/MD.0000000000015050. PMid:30921232. PMC6455907 30921232

[41] 41 Shi Y, Liu Z, Peng Z, Liu H, Yang K, Yao X. The diagnosis and treatment of Mullerian adenosarcoma of the uterus. Aust N Z J Obstet Gynaecol. 2008;48(6):596-600. 10.1111/j.1479-828X.2008.00914.x. PMid:19133051. 19133051

[42] 42 Juhasz-Böss I, Gabriel L, Bohle RM, Horn LC, Solomayer EF, Breitbach GP. Uterine Leiomyosarcoma. Oncol Res Treat. 2018;41(11):680-6. 10.1159/000494299. PMid:30321869. 30321869

[43] 43 Chen J, Liu X, Lan J, et al. Rhabdomyosarcoma in adults: case series and literature review. Int J Womens Health. 2022;14:405-14. 10.2147/IJWH.S352143. PMid:35370426. PMC8973688 35370426

[44] 44 American Cancer Society. Uterine sarcoma early detection, diagnosis, and staging. [place unknown]; 2022 [cited 2022 Oct 22]. Available from: https://www.cancer.org/content/dam/CRC/PDF/Public/8860.00.pdf

[45] 45 Varghese M, Bruland O, Wiedswang AM, et al. Metastatic mesenteric dedifferentiated leiomyosarcoma: a case report and a review of literature. Clin Sarcoma Res. 2016;6(1):2. 10.1186/s13569-016-0042-6. PMid:26913180. PMC4765132 26913180

[46] 46 Seagle BLL, Kanis M, Strohl AE, Shahabi S. Survival of women with Mullerian adenosarcoma: a national cancer data base study. Gynecol Oncol. 2016;143(3):636-41. 10.1016/j.ygyno.2016.10.013. PMid:27771166. 27771166

[47] 47 Zhang YY, Li Y, Qin M, Cai Y, Jin Y, Pan LY. High-grade endometrial stromal sarcoma: a retrospective study of factors influencing prognosis. Cancer Manag Res. 2019;11:831-7. 10.2147/CMAR.S187849. PMid:30697075. PMC6340498 30697075

[48] 48 Shankar V. Soft tissue; Skeletal muscle - Rhabdomyoma; Adult type rhabdomyoma. [place unknown]; 2021 [cited 2022 Oct 22]. Available from: https://www.pathologyoutlines.com/topic/softtissueadultrhabdomyoma.html

[49] 49 Shankar V. Soft tissue: Skeletal muscle - Rhabdomyoma; Genital type rhabdomyoma. [place unknown]; 2021 [cited 2022 Oct 22]. Available from: https://www.pathologyoutlines.com/topic/softtissuegenitalrhabdomyoma.html

[50] 50 Neha B, Manjunath AP, Girija S, Pratap K. Botryoid Rhabdomyosarcoma of the cervix: case report with review of the literature. Sultan Qaboos Univ Med J. 2015;15(3):e433-7. 10.18295/squmj.2015.15.03.022. PMid:26357564. PMC4554283 26357564

